# PD195 Variation In Decision-Making And Market Access Routes For Vaccines: Insights From Seven Countries

**DOI:** 10.1017/S0266462324004173

**Published:** 2025-01-07

**Authors:** Emily Gregg, Karina Watts, Charlotte Graham, Stuart Mealing

## Abstract

**Introduction:**

Quick and equitable market access to vaccines is a global priority. However, market access routes for vaccines are complex and differ from those for pharmaceuticals. Furthermore, there is variation in decision-making between countries due to local requirements. This work aimed to increase awareness of the key elements of these pathways and the stakeholders involved in European Union (EU) and non-EU countries.

**Methods:**

Pragmatic desk-based research was undertaken in November 2023 to explore key elements of the market access pathways for vaccines and how these differ between countries. Specifically, the countries of interest were Canada, England, France, Germany, Italy, Spain, and the USA. Where available, information was extracted about the key stages and stakeholders involved in the decision-making pathway as well as details about any post-licensing monitoring, the value assessment framework used, vaccine pricing, and the procurement process. In addition, examples of barriers to vaccine access were extracted. The key findings and between-country differences were summarized narratively.

**Results:**

National Immunization Technical Advisory Groups (NITAGs) were key stakeholders in all countries explored and had varying roles. The evidence requirements differed among countries, such as Germany’s requirement for economic and epidemiological modeling. The Vaccine Monitoring Platform coordinates studies for post-authorization monitoring of vaccines across EU countries. However, England is not part of this network and uses a national agency instead. Vaccine procurement and pricing also differed (e.g., France uses individual reimbursement, England uses national tendering, and Canada uses regional tendering). There was variation in vaccine pricing within the USA, depending on the healthcare provider. Barriers to vaccine access were well reported.

**Conclusions:**

These results can influence the market access strategy of vaccine developers to ensure rapid and equitable vaccine access across countries. Several between-country differences in vaccine market access routes were identified; for example, the role of NITAGs, evidence requirements, and post-licensing monitoring processes. Barriers to vaccine access have been reported in the literature, with some organizations providing recommendations to overcome these.

